# You Say You Want a Revolution: An Interview with Pat Brown

**DOI:** 10.1371/journal.pgen.1000560

**Published:** 2009-07-17

**Authors:** Jane Gitschier

**Affiliations:** Departments of Medicine and Pediatrics, Institute for Human Genetics, University of California San Francisco, San Francisco, California, United States of America

I have known Pat Brown for about two decades and he never ceases to amaze me. Over the years, I have heard him speak quite a few times, and on each occasion I can feel my jaw drop. What will he think up next?

**Image 1:**
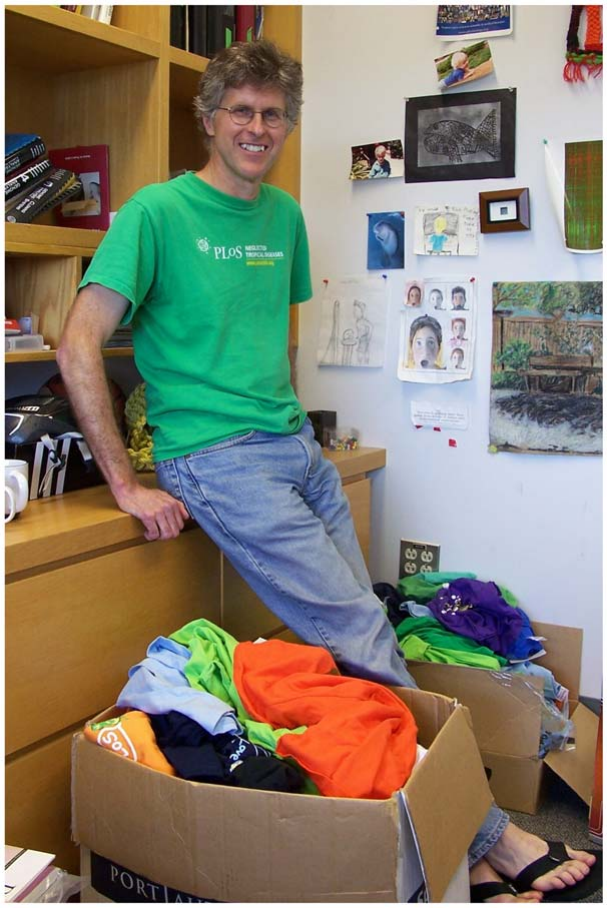
Pat Brown.

Pat (Image 1) is most frequently associated with the invention of microarrays and their use in studying gene expression, and he should be familiar to the readers of PLoS as a driving force behind open-access journals. But these are only two examples of his many successes, which span the worlds of topoisomerase, HIV integration, protein microarrays, and post-transcriptional regulation. Pat seems to have a brain in overdrive and the energy to match it. I was eager to tap into some of that electricity during the interview.

I met Pat in his office on the fourth floor of the Beckman building at Stanford, where he is a member of the Biochemistry Department. I arrived on a warm and fragrant spring afternoon to find Pat barefoot, wrapping up a grant submission, and obstructed by two large cardboard boxes of assorted PLoS T-shirts. On his door was a small poster: “Where would Jesus publish?”

I knew Pat had an atypical family story, so we started there. He is one of seven talented siblings, who were encouraged by their mother to think big and to make a contribution. His father's work led the family to spend four years in Paris, where Pat attended school in a quaint uniform of shorts with white hat and socks, and a second idyllic stint of four years in Taipei, in a neighborhood surrounded by rice paddies and water buffalo. In between, a Washington, D. C. suburb was home. Pat later discovered that his father did not work for the State or Defense Departments, as he had been led to believe, but rather the CIA, where he was an analyst.

We pick up the interview with discussion of an extremely fertile period in the early 1990s, when Pat was a new faculty member at Stanford and about to launch his work on DNA microarrays.

**Gitschier:** What was the initial thinking behind the microarray? I understand it had more to do with facilitating genotyping than expression measurements.

**Brown:** That's right. I was working on a scheme that had the ultimate aim of determining whole-genome genotypes of millions of people for linkage and association studies. It involved a biochemical method that we called “genomic mismatch scanning” for isolating the sequences that were identical between two genomes, and then mapping them by hybridizing to a physically ordered arrangement of the human genome.

At the time, you could map a cloned gene by FISH [fluorescence in situ hybridization] to metaphase chromosomes on slides, and that worked pretty well, but it wasn't scalable for the kind of experiments I was planning to do. You couldn't have Uta Francke, for example, just doing FISH after FISH experiment for all the sequences that we would be generating from this project.

I had a vision about how all this was going to go. I had sent a little blurb to Claire [Weinstock, an administrator at the Howard Hughes Medical Institute] outlining my plan. I used red and green dots to symbolize the microarrays, because I like that color combination.

**Gitschier:** You obviously aren't color blind. FISH uses just a single fluorescent probe, so why did you feel the need for a two-color, comparative system for the microarrays?

**Brown:** You need it to make reliable measurements. Kallioniemi had developed a method for complex probe comparative hybridization to metaphase chromosomes for looking at copy number variations. And that was precisely the rationale. If you were to just do a single probe hybridization, you would have very inhomogeneous patterns, only partially driven by the copy number changes themselves, but also by technical factors.

I had a small pilot grant from the NHGRI [National Human Genome Research Institute] to develop the genomic mismatch scanning method, and once Stan Nelson and I had that method worked out, I submitted a renewal application that included the microarrays.

I had a terrible experience with my renewal. In retrospect, I felt it was one of the best grant proposals I have written. And it got the worst priority score of any grant, not only of any grant I've ever written, but any grant I've ever SEEN.

**Gitschier:** Because it was too ambitious?

**Brown:** Yeah!

**Gitschier:** I can just imagine. They probably said each one of these specific aims is an entire grant.

**Brown:** The specific aims were 1. Take what we've already been doing biochemically [genomic mismatch hybridization] and make it work better and focus on the mammalian genomes instead of yeast. 2. Develop the microarray system from scratch. I said here's how I think we can do it, and it was pretty much exactly as we did start to do it. Aim 3 was development of statistical tools to take advantage of the high-resolution genome-wide genotype. Then I had an aim that we needed to start to put together the infrastructure to do this on a population basis. I had the idea that West Virginia was going to be a good place because it had the smallest fraction of the population moving in and out of the state. And there was yet another aim I can't remember.

I'm just trying to give you a sense of the weirdness of the grant. This is at a stage where all we had really done was to get this biochemical thing working in yeast.

**Gitschier:** So this was 1992.

**Brown:** Yeah, we submitted it in November 1992. And I thought, “This grant just totally rocks.” And then I got the little note-card back from the NIH [National Institutes of Health]. I saw my priority score: 344. I was just so totally deflated that I literally had to lie down on my office floor for ten minutes to regain my composure.

I got back in touch with them [NHGRI] and they said, “Just do aim 1 and resubmit the grant.” I did resubmit, but even in the grant proposal I said, “Following the advice, I'm doing this, but I think it's BAD advice and when I do get the grant I'm just going to be going ahead with other things I had proposed.” It was kind of stupid, but I was so pissed off that I just didn't want them to think that I was going to knuckle under.

**Gitschier:** And did you get that grant?

**Brown:** Yeah. It was much smaller, but I got it. Meanwhile I recruited Dari [Shalon] to start the microarray work, and that was a strange experience.

**Gitschier:** Tell me about it.

**Brown:** I went over to this building called CIS [Center for Integrative Systems], which is where they have a whole bunch of stuff set up, like the n-1 generation from micro-fabrication of the chip industry. So I thought, “This is where all the good stuff is for making very precise tiny things and patterning them.” So I went over there and just literally wandered around, asking people who I should talk to and I found Greg Kovacs, who was an MD/PhD neurologist. He was interested in building chips to be used for bionic people, for sensing impulses in nerves and controlling artificial limbs.

I got along well with him and told him what I had in mind to do and how I thought it could be done. I had it all laid out, using robotic printing. He kept wanting to make it a complicated electronic device. But I wanted it to be incredibly simple, and I wanted to use fluorescence read-out, not a circuit detecting changes in capacitance, for example, for a couple of reasons. One—I wanted to be able to do two colors and have the internal control, which you can't do with that direct sensing thing, and two—I didn't want some expensive high-tech thing that was going to be finicky.

That wasn't interesting to him, but he said, “I have a very good rotation student and this guy just wants to work on a project that is practical.” What it came down to was that he wanted something that he could use to start a company.

So I thought, “Fine, that's easy!” So I met with the guy [Dari] and told him the main thing I thought it would be good for commercially, which turned out not to be true, was for medical diagnostics. That you could build an array that would monitor the expression patterns in white blood cells. That these cells were acting like spies, that they were circulating to every part of the body—their whole purpose in life was to detect any kind of trouble and orchestrate a response, which involves a transcriptional program. So therefore, you should be able to take a drop of blood and look at what genes are expressed in white blood cells and figure out what they are seeing as an all purpose diagnostic.

Anyway, this is just an example of my attempts to lure him into the project. My pitch wasn't correct, but it had the effect of getting him to work on it. So Dari signs on. And I also had to give him clearance that if he developed something he could then take it and turn it into a commercial product.

**Gitschier:** Did he physically do this work in your lab?

**Brown:** Yes, but you had to live with Dari's personality. He was not an adorable guy.

Before Dari came in, I had this whole thing mapped out, an XY robot, we'd have stuff in micro-well plates and just dot spots. I wanted to use a system like a fountain pen because it's simple and robust—500-year-old technology. Dari wasn't too keen on that. He had a lot of ideas of his own; for example, he wanted to print on a linear tape which you'd scan by pulling it through some kind of reader. I never liked this idea at all, but he thought it might be a better system for scanning. There were a whole bunch of ideas, but finally we returned to the capillary printing thing. And that worked fine.

**Gitschier:** Had capillary printing been done before?

**Brown:** Not that I know of, but it must have been. The idea is so fundamental.

**Gitschier:** But you had this idea of a little fountain pen picking up a little bit of liquid, depositing it, going back and picking up something else.

**Brown:** Yes, the first model I had was from doing electron microscopy. When you pick up a grid, you hold it with these very fine tweezers and you put a little drop of stain on, and this annoying thing happens, not infrequently, that the little drop gets wicked up in the tweezers. And I had been doing some electron microscopy of some virus stuff. So literally the first things we used to do printing were tips of electron microscopy tweezers held together with a little epoxy to serve as our pen. So it was turning this annoying property of electron microscopy tweezers into something useful.

**Gitschier:** So where did you get the robotics? Did you build it?

**Brown:** Dari built the first one. Meanwhile, Joe [DeRisi] came to my lab to work on retrovirology stuff. I was trying to get him involved in the microarray stuff because I was trying to shift the center of gravity of the lab, but initially he wasn't buying it. But then, he was in the bay just down from Dari and eventually he got so annoyed that he felt he had to step in and do it better. Joe built the second and third generation printers. His robots are much better and fancier.

**Gitschier:** Eventually, though, Dari got something gridded.

**Brown:** Probably in less than a year. I have Dari's thesis somewhere up here. [Shows me.] Here is fluorescent DNA arrayed on the slide, just to show you can do it.

**Gitschier:** OK, so now we know we have DNA on slides, and we're going to do an experiment. The first one that is published is the Schena paper, which is an expression experiment and *Arabidopsis* at that. It has nothing to do with your original intent of genotyping. Tell me about that turn of events.

**Brown:** We [Stanford Biochemistry Department] have these yearly retreats. Dari was up to present from my group, and it might have been even in the same session as Mark Schena, who was in Ron Davis's lab. Mark talked about an experiment that he was trying to do. And this was the first talk about the Affy [Affymetrix] array.

**Gitschier:** So, just a sec. Somewhere in here, Affy is a player?

**Brown:** They had published a paper on putting optically encoded peptides on chips, a fantastic paper. Then we heard that they were working on doing this with oligonucleotides. And I knew they were at very early stages, able to make only 8-mers. Mark was trying to see if you could use those arrays to look at mRNA expression. But it didn't work at all—you got completely non-specific hybridization.

So immediately after he talked, and Dari had just given his talk, those two guys launched a collaboration, since we had microarrays that were clearly working. We had printed arrays with a bunch of different DNA sequences and different probes. Very high signal to noise.

Mark's idea was to take an isogenic strain overexpressing some transcription factor [and to look at the differences in expression profile compared to control], and I wanted to look at different parts of the plant, but it was all about a cute proof-of-principle experiment more than biology. It was a simple experiment because he had a bunch of cDNA clones and RNA isolated, just a matter of labeling it. Within a month or two, we had data for a paper.

The next interesting paper, as far as I was concerned, was the paper in which Joe was the first author. It was one of my all-time favorite papers. It was what we were going for from the get-go which is to be able to look at a whole genome.

**Gitschier:** But this thing with Schena and the expression tipped what kind of questions you were going to ask.

**Brown:** Right. One thing about the *Arabidopsis* experiment that made a big impression on me was that even by looking at a trivial number of genes, suddenly you could see a picture that is telling you the difference between a leaf and a root.

I just got very excited about it. You don't have to know anything about the mechanism at all. It made me switch gears and made me realize that actually, if you say that genetics is relating variation in the genome to variation in phenotype, there is more accessible variation in expression than there is in sequence, and there is more variation in phenotype between cells and tissues and organs than there is between people. From the standpoint of figuring out biology, that was probably the angle that was going to be more powerful.

What really tipped the balance was Joe's experiment on the diauxic shift, where a whole bunch of things became clear to me for the first time. How powerful it was to look for sets of genes that had correlated expression and how much information that carried about phenotype. Also, the fact that you could take a genome, in which only a third of the genes are annotated, and by looking at their patterns of expression, make pretty strong guesses about what they [the unannotated genes] may be doing. At that point, I thought, I still love genetics, but this is SO the low-hanging fruit! From the standpoint of doing exploratory experiments and discovering things—it was going to be way more fun.

People in the lab who were doing experiments just looking at gene expression patterns were just turning the crank. For them it was—have an interesting idea for a biological experiment, get data. Genotyping just couldn't compete any more.

**Gitschier:** I couldn't help but wonder, though, whether at some point the tail started wagging the dog. In other words, have you found yourself in a situation where you were too successful so that your time has been spent, maybe in these bigger collaborations…

**Brown:** You are so dead on!

**Gitschier:** …possibly to the detriment of your own creativity.

**Brown:** I feel that there is a lot of truth in that because I can get excited about just about anything! In the early days, I thought the best possible thing to do—and I told people in my lab to just roll with me on this one—was to seize any opportunity to get other people to provide us with the best possible samples. Because I thought that a large part of the way we were going to be able to make sense of every experiment we did was to collect a huge body of data. Data would have emergent properties that would make every little bit of it make sense. You could learn the dictionary of how to make sense of how the genome's language was used.

So, I was very promiscuous in terms of soliciting and when solicited, saying yes to collaborations. But what happened was that, very early on, I realized that we had not looked ahead enough. We had tons of things that could turn into papers, but a limited capacity to stop and write papers, especially when a collaborator would take some morsel that was very interesting and want to write a paper on it, but for me it was just one piece of the puzzle. We had experience in turning the data into a story, so we couldn't just hand off the data to people [without our help]. So that became a big drain.

I was a true believer in this and I think it was all very worthwhile. But there is a point at which you sort of know what the answer is going to look like, and where it is headed, and it's very important to see it through, but at that point for me, I'm ready to hand this off.

**Gitschier:** OK. May I ask about this grant application? I see some tissue staining [on the computer screen].

**Brown:** It's about developing methodology and software so that you could use a variety of different antibody stains and chemical stains in tissues, and for each one you have a quantitative value, for each pixel you have a vector of values, and you can cluster them the way you do for microarray data, to find things that are similar. And then you color code the images. And in this way, you can actually see specific cell types and pick up subtle quantitative differences in the staining.

One of the things I'm most interested in is that in most tissues, there is a lot more personality to the cells than you think. So I wanted to develop a way to look at a tissue section and say, these aren't just fibroblasts here, but actually 30 different kinds of cells.

This is going to be a really great diagnostic tool too. I want it to be really cheap and fast. You can stain a tissue with a few stains and the code takes almost no time to run. I showed this to Mark Krasnow and he said “We've been trying like hell to find a stain that showed a difference between these two cells,” and here we just threw on a few stains with absolutely no specificity for them, but the pixel clustering pulled out subtle quantitative differences in the staining and cleanly separated them. It's just like in FACS sorting [fluorescence-activated cell sorting].

**Gitschier:** This is very cool!

**Brown:** Ask me about my next big project.

**Gitschier:** OK, but first let's spend a minute on the genesis of PLoS.

**Brown:** I want to LITERALLY overthrow the scientific publishing establishment.

**Gitschier:** Do you want to say that again, only louder?

**Brown:** That is what I want to do. PLoS is just part of a longer range plan. The idea is to completely change the way the whole system works for scientific communication.

At the start, I knew nothing about the scientific publishing business. I just decided this would be a fun and important thing to do. Mike Eisen, who was a post-doc in my lab, and I have been brain-storming a strategic plan, and PLoS was a large part of it. When I started working on this, almost everyone said, “You are completely out of your mind. You are obviously a complete idiot about how publishing works, and besides, this is a dilettante thing that you're doing.” Which I didn't feel at all.

I know I'm serious about it and I know it's doable and I know it's going to be easy. I could see the thermodynamics were in my favor, because the system is not in its lowest energy state. It's going to be much more economically efficient and serve the customers a lot better being open access. You just need a catalyst to GET it there. And part of the strategy to get it over the energy barrier is to apply heat—literally, I piss people off all the time.

**Gitschier:** OK, Pat, with that, I think I'm ready to hear about the NEXT big project.

**Brown:** OK—I'm serious, and I'm going to do my sabbatical on this: I am going to devote myself, for a year, to trying to the maximum extent possible to eliminate animal farming on the planet Earth.

**Gitschier:** [Pause. Sensation of jaw dropping.]

**Brown:** And you are thinking I'm out of my mind.

**Gitschier:** [Continued silence.]

**Brown:** I feel like I can go a long way toward doing it, and I love the project because it is purely strategy. And it involves learning about economics, agriculture, world trade, behavioral psychology, and even an interesting component of it is creative food science.

Animal farming is by far the most environmentally destructive identified practice on the planet. Do you believe that? More greenhouse production than all transportation combined. It is also the major single source of water pollution on the planet. It is incredibly destructive. The major reason reefs are dying off and dead zones exist in the ocean—from nutrient run-off. Overwhelmingly it is the largest driving force of deforestation. And the leading cause of biodiversity loss.

And if you think I'm bullshitting, the Food and Agricultural Organization of the UN, whose job is to promote agricultural development, published a study, not knowing what they were getting into, looking at the environmental impact of animal farming, and it is a beautiful study! And the bottom line is that it is the most destructive and fastest growing environmental problem.

**Gitschier:** So what is your plan?

**Brown:** The gist of my strategy is to rigorously calculate the costs of repairing and mitigating all the environmental damage and make the case that if we don't pay as we go for this, we are just dumping this huge burden on our children. Paying these costs will drive up the price of a Big Mac and consumption will go down a lot. The other thing is to come up with yummy, nutritious, affordable mass-marketable alternatives, so that people who are totally addicted to animal foods will find alternatives that are inherently attractive to eat, so much so that McDonald's will market them, too. I want to recruit the world's most creative chefs—here's a REAL creative challenge!

I've talked with a lot of smart people who are very keen on it actually. They say, “You have no chance of success, but I really hope you're successful.” That's just the kind of project I love.

Do you feel like you are ridiculously optimistic?

**Gitschier:** Me? Yeah, sometimes. I have my share of wild ideas. But you—you want a revolution.

